# Challenges and Prospects for Eradication of *Helicobacter pylori*: Targeting Virulence Factors, Metabolism, and Vaccine Innovation

**DOI:** 10.3390/pathogens14070619

**Published:** 2025-06-21

**Authors:** Adrian Bakiera, Anita Solarz, Marika Kowalczyk, Halina Cichoż-Lach, Izabela Korona-Głowniak

**Affiliations:** 1 Department of Pharmaceutical Microbiology, Medical University of Lublin, 20-093 Lublin, Poland; 2 Stefan Cardinal Wyszynski District Specialist Hospital, 20-718 Lublin, Poland; 3 Department of Gastroenterology with Endoscopic Unit, Medical University of Lublin, 20-953 Lublin, Poland

**Keywords:** *Helicobacter pylori*, anti-*H pylori* treatment, antibiotic resistance, antibacterial agents

## Abstract

*Helicobacter pylori* is a Gram-negative bacterium that infects almost half of the global population and is linked to gastric conditions like peptic ulcers and gastric cancer, as well as other diseases such as neurological disorders, cardiovascular problems, and iron deficiency anemia. Its survival in the acidic stomach environment is due to virulence factors like urease, flagella, and adhesion proteins (BabA, SabA). Current treatments involve a combination of antibiotics (clarithromycin, metronidazole, amoxicillin, tetracycline) and proton pump inhibitors, but increasing antibiotic resistance, especially to clarithromycin and metronidazole, poses a major challenge. Resistance mechanisms include mutations in drug targets, efflux pump overexpression, and enzymatic degradation of antibiotics. This has prompted exploration of alternative therapies targeting bacterial processes like urease activity, biofilm formation, and metabolic pathways (energy production, amino acid synthesis, iron acquisition). Natural compounds, such as chitosan and plant extracts, show promise in combating *H. pylori* growth and virulence. Vaccine development is also ongoing, with DNA vaccines showing potential for broad immune responses. However, no vaccine is yet close to widespread clinical use.

## 1. Introduction

*Helicobacter pylori* is commonly found in the human gut. It is a microaerophilic, Gram-negative spiral-shaped microorganism [1]. It infects about half of the worldwide population, despite a reduction in the global prevalence of *H. pylori* infection over recent years [2]. In 1983, the role of *H. pylori* in gastrointestinal disorders such as peptic ulcer disease was proven by Barry J. Marshall and Robin Warren, which led them to win the Nobel Prize in Physiology or Medicine in 2005 [3]. Furthermore, the influence of *H. pylori* on carcinogenesis has been confirmed and in 1994 it was classified by IARC (International Agency for Research on Cancer) as a group 1 carcinogen [4], *H. pylori* infection is connected with gastric cancer and mucoid-associated lymphoid tissue lymphoma (MALT) but many researchers have linked *H. pylori* infection with diseases other than gastrointestinal disorders, including, among others, non-alcoholic fatty liver disease (NAFLD), neurological disorders like Alzheimer’s disease and Parkinson’s disease, iron deficiency anaemia, and cardiovascular diseases [5,6].

*H. pylori* owes its pathogenic ability to the release of several virulent factors. The invasion of bacteria into gastric epithelial cells and their colonization is enabled by outer membrane proteins, which include blood group antigen-binding adhesin (BabA), sialic acid-binding adhesin (SabA), outer inflammatory protein (OipA) [7], neutrophil-activating protein A (NepA), vacuolating cytotoxins (VacA), a cytotoxin-associated gene product (CagA), outer membrane vesicles (OMV), outer membrane protein (OMP), and high-temperature requirement A (HtrA) [8]. Other *H. pylori* OMPs include Helicobacter OMPQ (HopQ), Helicobacter OMPZ (HopZ) and the *H. pylori* outer membrane (Hom) family proteins (HomA, HomB, HomC, and HomD). However, before they can overcome the epithelial barrier, bacteria must survive in the unfavorable environment of the stomach. This is possible thanks to the helical shape of the bacteria and the presence of flagella, which enable penetration through the mucous layer. The characteristic agent of *H. pylori* that allows survival in an acidic environment is urease, an enzyme whose production constitutes 10% of the proteins produced by *H. pylori* [9,10]. All of the significant factors and metabolic pathways essential for invasion, infection, and bacterial life are targets for new drugs.

## 2. Current Treatment Strategies for *Helicobacter pylori*

In the process of *H. pylori* treatment, commonly known antibiotics with different mechanisms of action are used. Effective treatment is possible thanks to combination therapy, where at least two or three antimicrobial substances are combined with proton pump inhibitors (PPIs) and optionally in some treatment regimens with bismuth-containing compounds [11]. Among the antibiotics used in the eradication of *H. pylori*, amoxicillin is one of the safest and most effective, because of the low resistance level of this compound among *H. pylori* (≤10% resistance to amoxicillin and tetracycline) [12]. Amoxicillin is classified as β-lactam, which contains a four-membered β-lactam ring fused to a thiazolidine ring. The mechanism of action is based on inhibiting the synthesis of peptidoglycan, one of the most important cell wall components, as a result of which bacterial growth is blocked [13]. Tetracycline, a bacteriostatic antibiotic, inhibits protein synthesis by binding to the ribosomal 30S subunit. It also has a low resistance rate, which is why it is recommended for first-line treatment as a compound in bismuth quadruple therapy [14]. Fluoroquinolones, particularly levofloxacin, are also used in the treatment of *H. pylori*. The mode of action is based on promoting DNA damage by binding to two essential type II DNA topoisomerases, which leads to inhibition of DNA synthesis in bacterial cells [15,16]. Unfortunately, the usefulness of these antibiotics in first-line treatment is limited by toxicity, side effects like aortopathy, neuropathy, gastrointestinal ailments, and overuse leading to increased resistance to fluoroquinolones among bacteria including *H. pylori* [15,17]. Metronidazole is a drug approved for use against susceptible anaerobic bacteria, and it is also commonly used in various treatment regimens to eradicate microaerophilic *H. pylori*; however, the mechanism of action against *H. pylori* is not well understood [18]. Clarithromycin is a macrolide that affects bacteria by interfering with protein synthesis in bacterial cells through binding to the ribosome subunit [19].

These antibacterial agents are the most often used in *H. pylori* treatment, notwithstanding that in various treatment regimens, rifabutin is also considered [11]. Apart from antibiotics, it is necessary to include other medications in the treatment, as follows:

(I) Proton pump inhibitors (PPIs) lead to more alkaline pH in the gastric mucosa, necessary to obtain optimal bioavailability of the acid-susceptible antibiotics, by inhibiting the gastric acid pump (H+/K+-ATPase). Moreover, PPIs can affect *H. pylori* growth by directly inhibiting it, thereby supporting its eradication [20]. Several proton pump inhibitors are available; rabeprazole or esomeprazole 20 to 40 mg twice daily is recommended. Rabeprazole is preferred because it undergoes primarily non-enzymatic metabolism, with minimal involvement of the genetically variable enzyme CYP2C19. This results in more consistent acid suppression that is less affected by patients’ genetic differences [20].
(II) Bismuth subsalicylates are part of some treatment regimens due to their anti-inflammatory and bactericidal properties [21]. However, bismuth mechanism’s of action on *H. pylori* is still not fully understood, notwithstanding research suggesting that bismuth can cause several abnormalities in bacterial cells by inhibition of enzymes, i.e., urease, catalase, and lipase, binding to the bacterial wall and periplasmic space and causing bacterial cell damage and inhibiting adherence to the surface of epithelial cells. Moreover, bismuth subsalicylates show advantageous gastroduodenal effects, for example, by protecting gastric mucous from peptic luminal degradation [22].

Progressive antibiotic resistance and difficulties in *H. pylori* eradication have forced experts to update outlines for treatment and eradication. Current recommendations in the 6th edition of the Maastricht/Florence Consensus published in 2021 advise a treatment regimen customized to the local prevalence of antibiotic resistance [11]. Bismuth quadruple therapy (BQT) is recommended as first-line therapy in the Maastricht VI/Florence Consensus and also by the American College of Gastroenterology and Toronto Consensus [11,23,24]. BQT therapy is based on bismuth and a combination of two antibiotics, tetracycline and metronidazole, with PPI. Clarithromycin triple therapy including PPI, clarithromycin, and amoxicillin for a duration of 14 days is also recommended as first-line therapy but only in regions with proven low clarithromycin resistance and local effectiveness. In regions with high or unknown clarithromycin resistance BQT is mostly recommended for first-line treatment [11].

The Maastricht VI/Florence Consensus report recommends considering culture and antibiogram before implementing second-line therapy. Second-line therapy, introduced in case of failure of first-line treatment, should not be based on the same regimen as the previous therapy; bismuth-containing quadruple therapy or a levofloxacin-containing triple therapy are recommended [11].

## 3. Difficulties in *H. pylori* Treatment

Despite the substantial progress in understanding *H. pylori* pathogenesis, significant gaps remain regarding the genomic, populational, and metabolic heterogeneity of this bacterium, particularly concerning regional and intragastric variability of virulence factors. Such diversity can affect not only the course of infection but also the risk of developing severe gastroduodenal diseases, including cancer. Differences in the distribution of virulence genes such as *cag*A and *vac*A have been observed among populations from various geographical regions, and heterogeneity has also been reported within different areas of the stomach in the same host. The genetic and phenotypic variability of *H. pylori* strains may have direct implications for treatment outcomes [25,26].

Marked differences in the distribution of virulence genes such as *cag*A and *vac*A have been reported across distinct geographical regions. For example, an analysis of Chilean and Cuban *H. pylori* strains revealed a higher prevalence of *cag*A-positive strains with multiple EPIYA-C motifs in Chile, a pattern linked to more severe histological alterations in the gastric corpus [27]. Similarly, variations in *H. pylori* genotype-host co-evolution are thought to underlie region-specific gastric cancer risks, as discussed in studies focusing on differences between African, Asian, and Latin American populations [28].

Intragastric heterogeneity of *H. pylori* virulence factors has also been documented. Recent data from Peruvian patients indicated variable detection rates of *cag*A and *vac*A genes depending on the gastric region sampled, with potential implications for disease severity and diagnosis [29]. Such localized variation may partly explain the inconsistencies often observed between biopsy sites within the same individual.

Further studies support the influence of both bacterial and host factors on the pathogenic potential of *H. pylori*. A broad assessment of *vac*A allelic variation across continents underlined the association of certain genotypes (e.g., *vac*A *s1/m1*) with increased risk of ulcers and gastric malignancy, particularly in East Asian strains [25]. Moreover, the interplay between host genetic ancestry and bacterial genotype appears to modulate disease outcome, as strain–host mismatches have been linked with elevated gastric pathology risk in populations with admixed ancestry [30].

These observations underscore the importance of a comprehensive, regionally nuanced approach to *H. pylori* research, considering both inter-populational differences and the microgeographical variability within the gastric niche itself.

Treatment of *H. pylori* infection is empirical and the main problem is antibiotic resistance, which is why cure rates are not satisfactory and eradication is ineffective in about 20% of patients. *H. pylori* strains invulnerable to clarithromycin were listed by the World Health Organisation (WHO) as one of the 12 priority pathogens requiring new therapeutic agents. Antibiotics aimed at *H. pylori* strains with resistance defined by culture and antibiogram seem to be the best solution. Unfortunately, targeted therapy is complicated, time-consuming, and hard to implement due to the necessity of invasive collection of specimens and hard-to-obtain culture. Apart from increasing antibiotic resistance, treatment of *H. pylori* requires good compliance from the patient, and the side effects of drugs used in eradication can discourage patients from cooperating and following the doctor’s recommendations [30,31].

## 4. Antibiotic Resistance in *Helicobacter pylori*

The prevalence of antibiotic resistance in *H. pylori* varies considerably between different regions worldwide. While countries in Europe and North America tend to report lower resistance rates, regions like Southeast Asia, South America, and parts of Eastern Europe show significantly higher levels of resistance. For instance, recent studies show that clarithromycin resistance exceeds 40% in parts of Southeast Asia, while in many European countries and the USA, resistance rates are typically below 20%. Metronidazole resistance follows a similar pattern, with rates as high as 40% reported in developing countries, compared with much lower rates in high-income countries [32]. On the other hand, amoxicillin resistance is still relatively low worldwide, which has allowed it to remain a key component of first-line treatment regimens, though this could change as resistance rates slowly rise [33].

Geographic differences in resistance can be attributed to various factors, including the frequency of antibiotic use, healthcare practices, and *H. pylori* infection rates. For example, in Southeast Asia, the widespread and often inappropriate use of antibiotics has accelerated the development of resistance, while regions like Japan have been more successful in controlling resistance, thanks to strict antibiotic stewardship programs and lower overall infection rates [34]. Meanwhile, in Latin America, similar trends to those seen in Southeast Asia are evident, with high clarithromycin resistance rates making standard treatments less effective [35].

## 5. Primary Pathways of Antibiotic Resistance in *H. pylori*

Bacteria have developed various mechanisms of drug resistance (Table 1), the main pathways of which are discussed below.

### 5.1. Genetic Mutations

A major factor contributing to antibiotic resistance in *H. pylori* is the occurrence of mutations in key bacterial genes. These mutations often affect genes that are responsible for important cellular processes like protein synthesis or cell wall construction. For example, mutations in the 23S rRNA (A2142G or A2143G) gene are primarily responsible for the resistance of *H. pylori* to clarithromycin, while mutations in 16S rRNA determine resistance to tetracycline. These changes in the gene structure alter the antibiotic’s ability to bind to its target, making the treatment ineffective [36,37]. Mutations in the *frxA* or *rdxA* genes contribute to metronidazole resistance, while fluoroquinolone resistance is determined by *gyrA* mutations [38,39].

### 5.2. Efflux Pumps

Another mechanism by which *H. pylori* can resist antibiotics is by utilizing efflux pumps, which are proteins that actively pump antibiotics out of the bacterial cell before they can reach high enough concentrations to be effective. Efflux pumps are involved in resistance to various classes of antibiotics, including tetracyclines, fluoroquinolones, and macrolides. By expelling antibiotics from the cell, these pumps prevent the drugs from achieving the levels necessary to inhibit bacterial growth and replication [40,41]. This mechanism is particularly troublesome in regions where antibiotics are overused, contributing to the rise of multidrug-resistant *H. pylori* strains [37].

### 5.3. Enzymatic Degradation

Some strains of *H. pylori* produce enzymes capable of breaking down antibiotics before they can exert their effects. For instance, the bacterium produces enzymes like nitroreductases, which activate metronidazole by converting it into its toxic form. Mutations in *rdxA* that affect the production or activity of these enzymes can prevent metronidazole from being activated, resulting in resistance [42]. Other enzymes—beta-lactamases—can degrade beta-lactam antibiotics like amoxicillin, rendering them ineffective, which has been observed in some highly resistant *H. pylori* [43].

### 5.4. Alteration of the Antibiotic Target Sites

A common mechanism of resistance in *H. pylori* involves changes to the sites where antibiotics normally bind. For instance, alterations in the penicillin-binding proteins (PBPs) of the bacterium can prevent beta-lactam antibiotics (amoxicillin) from binding effectively to their targets, thus reducing their effectiveness [44].

**Table 1:** *H. pylori* antibiotic resistance mechanisms [33,37,40].

Antibiotic	Resistance Mechanism	Involved Genes/Mechanisms
Amoxicillin	Decreased affinity of penicillin-binding proteins (PBPs)	Mutations in PBP1 or PBP2
	Overexpression of efflux pumps	*hp1165*, *hefA*
Tetracycline	Modification of ribosomal binding site	Mutations in 16S rRNA
	Overexpression of efflux pumps	*hp1165*, *hefA*
Rifamycins	Mutations in the β subunit of RNA polymerase	Mutations in *rpoB* (codons 525–586)
Levofloxacin	Mutations in DNA gyrase (QRDR region)	Mutations in *gyrA*
	Mutations in *gyrB*	Less common than *gyrA*, but may enhance resistance
	Overexpression of efflux pumps	*hp1165*, *hefA*
Metronidazole	Loss of function of reducing enzymes	Mutations in *rdxA* (stop codons, deletions)
	Mutations in *frxA* (NADPH-dependent reductase)	Stop codons, deletions
	Overexpression of efflux pumps	*hp1165*, *hefA*
Clarithromycin	Modification of ribosomal binding site	Mutations in 23S rRNA, mainly A2143G, A2144G, A2142G, A2142C
	Overexpression of efflux pumps	*hp1165*, *hefA*

## 6. Mechanism of Antibiotic Resistance in *H. pylori*

### 6.1. Resistance to Clarithromycin

Clarithromycin, a macrolide antibiotic, has been a cornerstone of first-line therapy for *H. pylori* eradication. However, resistance to clarithromycin has become increasingly prevalent, particularly in regions with high levels of antibiotic use [45]. In Southeast Asia clarithromycin resistance rates can exceed 40%, often due to the widespread and inappropriate use of antibiotics. In contrast, resistance rates are generally lower in North America and Western Europe, typically ranging between 10% and 20% [46]. In Latin America, resistance rates are often higher, with some countries like Brazil and Argentina reporting rates of over 30% [35]. The increasing resistance to clarithromycin poses a significant challenge to its effectiveness.

The most common mechanism of resistance in *H. pylori* is the presence of mutations in the V domain of 23S rRNA gene, particularly at positions: A2143G, A2144G, A2142G or A2142C. These mutations alter the binding site of clarithromycin on the bacterial ribosome, impairing its ability to inhibit protein synthesis and thereby allowing bacterial survival [37,47,48]. Resistance is further exacerbated by the action of efflux pumps—membrane-bound transporters that actively remove the antibiotic from the bacterial cell. This reduces the concentration of clarithromycin inside the cell, limiting its therapeutic effect [40]. Moreover, there is an interdependence between efflux pump activity and mutations in resistance genes. This shows that both mechanisms can act synergistically to increase resistance [41]. The global spread of clarithromycin-resistant *H. pylori* strains has led to reconsideration of the efficacy of standard treatment regimens, particularly in areas with high resistance [49]. Options such as bismuth-based quadruple therapy or levofloxacin-based combinations are increasingly recommended in these regions [33].

### 6.2. Resistance to Metronidazole

Metronidazole is a nitroimidazole antibiotic commonly used in the treatment of *H. pylori* infections, especially when resistance to other antibiotics is present. However, it also exhibits high resistance rates in certain parts of the world. Resistance to metronidazole is particularly high in regions such as Southeast Asia, South America, and Eastern Europe, where resistance rates can exceed 70% in some areas [33,50]. This is largely due to the widespread use of metronidazole for treating a variety of infections, including parasitic diseases, and as a broad-spectrum antimicrobial agent. In contrast, metronidazole resistance is generally lower in Western countries, although this is increasing as *H. pylori* becomes more resistant to multiple antibiotics. Moreover, levels of metronidazole resistance in Japan remain exceptionally low as a result of the restricted antibiotics policy [51,52].

Resistance to metronidazole occurs primarily due to mutations in the *rdxA* or *frxA* genes that encode for the NADPH nitroreductase enzyme. It is involved in activating metronidazole within the bacterium. When these enzymes are mutated or absent, metronidazole is not converted into its toxic form, and the drug becomes ineffective against the bacteria [41,42]. In addition to these mutations, overexpression of efflux pumps plays a pivotal role in the resistance mechanism. These pumps expel metronidazole from the bacterial cell, preventing the drug from accumulating to toxic levels. This mechanism is particularly common in *H. pylori* strains isolated from areas where metronidazole is frequently used, such as in the treatment of other infections [39,40]. High metronidazole resistance rates in many regions complicates *H. pylori* treatment, especially when it is used as part of a triple therapy regimen. In regions with high resistance, clinicians often recommend alternative therapies, such as bismuth-based quadruple therapy, which involves the use of tetracycline or levofloxacin [32,53].

### 6.3. Resistance to Amoxicillin

Amoxicillin, a beta-lactam antibiotic, is another crucial component of *H. pylori* treatment which is often used as part of first-line therapy. Fortunately, resistance to amoxicillin remains relatively low, typically under 10%, and is considered rare in most parts of the world [33]. This makes amoxicillin a reliable choice for combination therapy. However, while resistance to amoxicillin is minimal, the rising resistance to other antibiotics, such as clarithromycin and metronidazole, is making first-line regimens less effective in some areas [33,54]. Resistance to amoxicillin is rare but can occur due to mutations in penicillin-binding proteins (PBPs), specifically PBP1 and PBP2. These proteins are responsible for cross-linking the bacterial cell wall during cell division. Mutations in these proteins alter their binding affinity for beta-lactam antibiotics, including amoxicillin, thereby reducing the drug’s effectiveness [55,56]. Continuous monitoring for resistance is essential to ensure that amoxicillin can continue to be used effectively in treatment regimens, especially as *H. pylori* resistance patterns evolve [57].

### 6.4. Resistance to Levofloxacin

Levofloxacin, a fluoroquinolone antibiotic, is commonly used as part of second-line therapy in areas with high resistance to first-line antibiotics. Resistance to levofloxacin is growing worldwide, with particularly high rates in Southeast Asia and Europe. In certain regions, resistance can exceed 30%, making levofloxacin less effective in second-line treatments [58]. The rise in resistance is often linked to the overuse of fluoroquinolones in treating respiratory and urinary tract infections, contributing to the spread of resistant *H. pylori* strains [59].

The increasing resistance to levofloxacin underscores the need for ongoing surveillance of resistance patterns to ensure that second-line therapies remain effective. In regions with high resistance, alternative antibiotics such as rifabutin or tetracycline may be considered as part of second-line or third-line therapy [49,58]. Resistance to levofloxacin is largely attributed to mutations in the genes encoding the enzymes DNA gyrase (*gyr*A or *gyr*B), which are the primary targets for fluoroquinolones. These mutations alter the antibiotic’s binding sites, preventing it from effectively inhibiting DNA replication and repair [38,60].

The activity of efflux pumps may further contribute to levofloxacin resistance by reducing the intracellular concentration of the drug. This additional resistance mechanism complicates treatment strategies, especially in regions with high levels of fluoroquinolone resistance [37,40]. In light of growing resistance, the use of levofloxacin should be carefully monitored, and alternative treatment strategies should be considered in high-resistance areas [61,62].

### 6.5. Resistance to Tetracycline

Tetracycline remains a good option in some combination therapies for *H. pylori* infections, particularly in countries where resistance to other antibiotics is high. While tetracycline resistance is generally not a major concern, its use is often limited by its availability and the complexity of multi-drug regimens. Resistance rates are typically low, often under 2%, making tetracycline a valuable component of combination therapies, particularly in areas with high resistance to other first- and second-line antibiotics [63,64]. Of note, increasing tetracycline resistance has been observed in regions, which may compromise the efficacy of bismuth-based quadruple regimens where this antibiotic is a key component. Recent data report a resistance rate of 18.25% in Italy and 22% in Iran, highlighting the threat of reduced treatment success even in second-line or rescue therapies if this trend continues [65,66]. Its role in bismuth-based therapies makes it an important option, particularly in regions with high resistance to clarithromycin and metronidazole [67]. Tetracycline resistance in *H. pylori* is primarily mediated by mutations in the 16S rRNA gene that affect the binding site of tetracycline [68]. Also, efflux pumps are associated with resistance to tetracycline, particularly those encoded by the *hp1165* or *hefA* genes, which pump tetracycline out of the bacterial cell, reducing the drug’s intracellular concentration and making it less effective [61].

### 6.6. Resistance to Rifamycins

Rifamycins are a group of antibiotics that inhibit RNA synthesis in bacteria by binding to their polymerase. This group includes rifampicin and rifabutin, among others [69]. Resistance to rifamycins in *Helicobacter pylori* globally remains very low (below 1%), with a very high eradication rate of up to 90%. Their use is recommended in cases of failure of first- or second-line therapy due to potential side effects [70]. *H. pylori* resistance to rifamycin may be due to the action of efflux pumps, which transport the antibiotic outside the bacterial cell. This allows bacteria to effectively avoid the toxic effects of rifamycin, reducing its concentration inside the cell [40]. Mutations in the *rpoB* gene also determine resistance to rifamycins in *H. pylori*. Mutations typically located in codons 525–586 are associated with high resistance to this group of drugs [71]. Nevertheless, rifamycins remain a good alternative as a third- or fourth-line therapy.

## 7. Potential New Drug Targets for *Helicobacter pylori*

**Figure 1:**
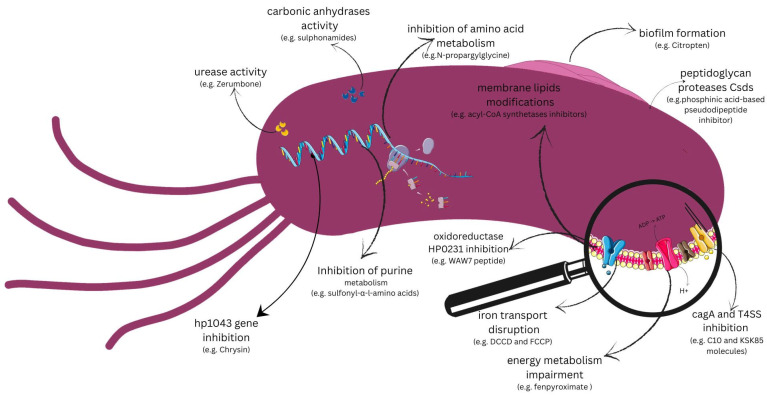
Potential targets for anti-*Helicoabcter pylori* agents.

With a growing understanding of *H. pylori*’s unique physiology and advancements in antibacterial drug discovery, there is strong potential for developing novel therapeutics to effectively treat *H. pylori* infections. A new approach to preventing virulence rather than bacterial viability is more advantageous because it is not limited by resistant strains and does not disrupt the gut microbiota. The current trend is to look mainly for antibacterial substances that affect various metabolic pathways of the bacterial cell or target its crucial genes and enzymes (Figure 1). Moreover, several virulence factors are easily accessible to extra-cytoplasmic molecules and their structure has been explained; so, novel inhibitors could be designed and investigated in silico using molecular docking techniques [72].

### 7.1. Targets Pointed to Virulence Factors

#### 7.1.1. Urease

Urease is an essential enzyme and virulence factor of H. pylori, which enables colonization of gastric mucosa and persistent infection of *H. pylori* in the stomach. The action of urease is nickel-dependent and is based on increasing pH in the gastric mucus layer by urea hydrolysis to ammonia and carbon dioxide [73]. This fact is cited by some researchers considering a nickel-free diet as an advantageous addition to standard triple therapy. Improvement in the eradication rate of *H. pylori* is caused by reduced activity of urease, leading to higher exposure of the bacterium to gastric acid, which significantly decreases its chances of survival [74]. Regarding *H. pylori*, mention has been made of its noncatalytic role in the bacterial invasion process. The role of urease has also been demonstrated in modulating the host immune response, stimulating neutrophil and monocyte chemotaxis, inducing pro-inflammatory cytokines, and binding with class II major histocompatibility complex MHC receptors to induce apoptosis in gastric epithelial cells [75]. Undeniably, the role of urease in the invasion process and survival is the reason why researchers are focusing on the development of drugs with urease inhibition as a point of action. Potential compounds able to inhibit urease include plant-origin substances (e.g., Zerumbone), which suppress urease activity by forming it into bigger particles such as dimers, trimers, or tetramers [76]. Compounds based on the structure of catechol can also present antiureolytic properties. They inactivate urease by irreversibly modifying cysteine residues at the entrances to enzymatic active sites. In studies from 2023, 2-(3,4-Dihydroxyphenyl)-3-phosphonopropionic acid appeared as the most promising antiureolytic compound [77]. Currently, both of these substances need more research on their activity *in vivo*. It may be interesting to use chitosan—a polysaccharide naturally found in shrimps or crab shells—for treatment of *H. pylori* infection. Chitosan is partially deacetylated chitin that can inhibit the production of urease by *H. pylori* and block its growth. In addition, chitosan also exhibits these properties in combination with antibiotics used in the standard treatment of *H. pylori*—amoxicillin, tetracycline, and metronidazole [78]. This preliminary research on natural substances is very promising, but their mode of action needs to be better understood.

#### 7.1.2. Carbonic Anhydrases

Carbonic anhydrases (CAs) are enzymes with zinc in their active center that catalyze the reversible reaction of the conversion of carbon dioxide into bicarbonate. *H. pylori* possess two of eight so-far specified classes of carbonic anhydrases (α, β, γ, δ, ζ, η, θ, ι): α-carbonic anhydrase (HpαCA) and β-carbonic anhydrase (HpβCA) [79]. α-CAs occur in periplasm whereas β-CAs occur in the cytosol and on the cytosolic side of the inner membrane; despite their different roles they both allow bacterium cells to persist in the acidic, unfavorable environment of the stomach by maintaining optimal periplasmic and cytoplasmic pH and taking part in urease-dependent response to acidity [80,81]. Therefore, substances capable of inhibiting CA are promising therapeutic possibilities. It was discovered that human CA inhibitors used for years in conditions such as glaucoma and ulcers and as diuretics are also active against *H. pylori* CAs. These inhibitors—sulphonamides—mimic the catalytic transition state of CO_2_ conversion. From the group of the sulphonamides, ethoxzolamide seems to have the best activity against *H. pylori*, but it is not in widespread use due to its quite weak antibacterial activity in comparison to antibiotics used in the treatment of *H. pylori* infection [82]. Another substance commonly used as an anti-ulcer drug—famotidine—is also able to inhibit several human and *H. pylori* CAs. Apart from being an antagonist of the histamine H2 receptor, famotidine inhibits CAs present in *H. pylori* by creating a binding. These functions may be used in the future to design new anti-microbial drugs or substances supporting the treatment process [83]. Inhibiting bacterial CAs is promising for the eradication of *H. pylori*; therefore, it seems necessary to seek new substances active against CAs.

#### 7.1.3. Biofilm Formation

Biofilm is a unique formation consisting of adherent aggregates of bacterial cells immobilized by an extracellular polymeric substance (EPS). These modes of life ensure bacteria resistance to antibiotics, unfavorable external factors, host immune defenses, and protection by desiccation [84]. *H. pylori* creates a biofilm in the human gastric mucosa, which leads to chronic, difficult-to-treat infection. Moreover, *H. pylori* isolates able to create biofilm are more often resistant to clarithromycin than those that do not produce [85,86]. Formation and maintenance of biofilm is favored by adhesion proteins secreted by *H. pylori* (e.g., OipA and SabA), chemotaxis proteins that enable flagellar rotation participating in biofilm initiation, and enzymes like arginase or urease that ensure products or remove unfavorable components that provide an optimal environment for biofilm development. Additionally, secretions of EPS (extracellular polysaccharide) and eDNA (extracellular DNA) as components of biofilm ensure its proper, stabilized structure, while expressing quorum sensing molecules is necessary for communication by cells organized in biofilm structure [87]. LuxS enzyme synthesizes autoinductor-2 (AI-2), *H. pylori*’s only known quorum-sensing molecule, with production peaking during mid-exponential growth. AI-2 via LuxS is the central quorum-sensing system in *H. pylori*, regulating both the formation and dispersal of biofilms [88]. Quorum quenching interference, for example, enzymatic degradation of AI-2, may be a promising strategy for controlling *H. pylori* biofilms. Enzymes like AiiA lactonase from *Bacillus licheniformis* degrade AI-2, reducing the biofilm’s biomass, thickness, exopolysaccharide production, and urease activity *in vitro* [88]. Flagellar motility and chemotaxis are directly linked to quorum sensing and may serve as additional therapeutic targets. AI-2 influences the expression of flagellar genes (*flaA, flgE, motA/motB, flhA*) and motility. Through modulating motility, AI-2 also indirectly affects biofilm initiation and spatial organization [89]. N-Acetylcysteine (NAC), a mucolytic compound, has been known for some time as an anti-biofilm agent capable of inhibiting *H pylori* biofilm formation and also destroying already developed biofilms. The exact mechanism of NAC action remains unclear, but it may act by disrupting disulfide bonds that link glycoproteins in the mucous [85,90]. However, larger studies on NAC application in treatment regimens are needed. Anti-biofilm producing properties are often sought in plant origin substances like *Citrus sinensis* L. Extracts from *C. sinensis* leaves include coumarins, bergapten, xanthotoxin, and citropten, among which citropten is a promising anti-*H. pylori* agent with quite promising activity. One of described mechanism of action of citropten is the inhibition of *H. pylori* growth and biofilm [91]. Other studies on plant extracts as anti-biofilm substances are at an early stage of research, but designing drugs based on inhibition of biofilm production may become a popular research direction in the future.

#### 7.1.4. *hp1043* Gene

The *hp*1043 gene, also known as *hsr*A, present in the *H. pylori* genome is the crucial gene for cell viability. HsrA (homeostatic stress regulator) encodes the OmpR-like “orphan” response regulator that takes part in coordinating the expression of genes related to the bacteria’s central metabolism and virulence, with genes responsible for cell division and bacterial growth. Moreover, *hsr*A is involved in mediating the response to oxidative stress. The key role of this gene explains its potential as a new drug target to eradicate *H. pylori* by inhibiting its expression [92,93]. Recent studies on the natural flavonoids apigenin, chrysin, kaempferol, and hesperetin revealed their bactericidal activities against *H. pylori* with different strengths. Their mode of action *in vitro* is based on inhibiting the DNA binding activity of *hsr*A. What is more, some of them, like chrysin, demonstrate synergistic effects with currently used antibiotics including clarithromycin and metronidazol [93]. Hesperetin seems to show more effect on *H. pylori* cells than inhibiting only the *hsr*A gene, but it can also downregulate urease subunit proteins, genes constituting flagella, and adhesion-related genes, and it downregulates genes encoding CagA and VacA, virulence factors that together prevent *H. pylori* from successfully colonizing and surviving in human gastric epithelial cells [94]. There is a need for further, in-depth studies on flavonoids as anti-Helicobacter agents, but their potential is promising.

#### 7.1.5. Peptidoglycan Proteases

Numerous proteases, named cell shape-determinant genes or Csds, act on the peptide chains of peptidoglycan and can have a significant role in determining *H. pylori* cell shapes. For example, Csd4 was investigated *in vitro* as a novel drug target. A phosphinic acid-based pseudodipeptide inhibitor was designed to act as a tetrahedral intermediate analog against the Csd4 enzyme. It was shown that highly polar compounds were capable of crossing the outer membrane and altering *H. pylori* cell shapes, presumably by inhibiting cell shape determinant proteases [95].

#### 7.1.6. CagA Toxin and Type IV Secretion System (T4SS)

The *cag* genes encode proteins that form a contact-dependent secretion system, enabling the bacterium to transfer the effector molecule CagA into host cells. Once inside, CagA is linked to severe gastritis and carcinoma. Additionally, functional T4SSs and CagA contribute to the stimulation of interleukin (IL)-8, a key factor in chronic inflammation. A small compound that blocked CagA transport was tested. The compound efficiently inhibited the function of a single component of the *H. pylori cag* T4SS, *Cagα*, and thereby blocked the biological activity of the system as a whole. Preliminary evaluation studies *in vitro* indicated that it mediated the reduction of pathogenic effects of *H. pylori* in mice [96]. Additionally, two synthetic small molecules have been identified for their ability to disrupt T4SS-dependent processes in various bacterial pathogens, including *H. pylori*. One compound inhibited the biogenesis of the pilus appendage associated with the cag T4SS to deliver the oncogenic effector protein CagA and peptidoglycan into gastric epithelial cells, while the second interfered with cag T4SS activity without affecting pilus assembly [97]. Inhibition of the T4SS system in *cagA*-positive patients could be a promising treatment strategy.

#### 7.1.7. HP0231 Oxidoreductase

Recent studies have explored potential inhibitors targeting HP0231, a unique dimeric oxidoreductase in *H. pylori* involved in disulfide bond formation. One of the approaches utilized in silico-designed small peptides to inhibit HP0231 activity. Specifically, the peptide WAW7, containing a single cysteine residue, demonstrated strong inhibitory effects by forming a disulfide bond with the active site cysteine (C159) of HP0231, effectively blocking its function. Another peptide, WAW8, which includes two cysteine residues, exhibited weaker inhibition, probably due to its susceptibility to oxidation by HP0231, reducing its availability as an inhibitor [98]. These findings suggest that designing peptides or small molecules capable of selectively binding to HP0231 active site cysteine residues may serve as a viable strategy for inhibiting its oxidoreductase activity, potentially attenuating *H. pylori* virulence.

### 7.2. Targets Pointed out on Metabolic Pathways

#### 7.2.1. Energy Metabolism in *H. pylori*

Energy metabolism in *H. pylori* plays a crucial role in its ability to survive in an acidic stomach environment. Unlike many other bacteria, *H. pylori* relies on a variety of unique pathways for energy production, including oxidative phosphorylation. Inhibitors such as diflumetorim, fenpyroximate, or fenazaquin targeting the *H. pylori* respiratory complex I quinone-binding pocket have shown selective activity against *H. pylori* while not affecting other bacteria [99].

One key target is cytochrome c oxidase (CcoN), a central enzyme in the electron transport chain that enables aerobic respiration. Compounds such as sulfur sulfite can disrupt CcoN leading to reduced ATP synthesis, which increases oxidative stress and leads to bacterial death [100]. Another critical enzyme in *H. pylori* energy metabolism is fumarate reductase, involved in anaerobic respiration. Inhibitors targeting fumarate reductase, namely 2-thenoyltrifluoroacetone, could impair the bacterium ability to adapt to low-oxygen environments, a key feature of *H. pylori* survival in the gastric mucosa [101].

Recent studies have also identified the arginine deiminase system (ADS) as a potential target. The ADS pathway enables H. pylori to survive under oxygen-limited conditions by converting arginine to ammonia and carbon dioxide, producing energy. For example, inhibition of ADS by a-difluoromethylornithine can improve host immune response and kill bacteria [102].

#### 7.2.2. Inhibition of Amino Acid Metabolism

Amino acid metabolism is crucial for *H. pylori*’s survival and virulence. Unlike many other organisms, *H. pylori* has a limited ability to synthesize several essential amino acids and must gain them from its host. Targeting amino acid biosynthesis pathways offers a promising strategy to improve *H. pylori* eradication [103].

The biosynthesis of proline and the deamination of asparagine and glutamine play an important role in the bacterial cell stress response and membrane integrity [103,104]. Inhibition of key enzymes such as proline dehydrogenase by N-propargylglycine and its analogs, which catalyze the first step in proline catabolism, has been shown to significantly impact *H. pylori*’s ability to colonize the gastric mucosa [105,106].

#### 7.2.3. Fatty Acid Metabolism

Fatty acid metabolism is essential for maintaining *H. pylori* cellular membrane structure and function [107]. The bacterium’s ability to synthesize fatty acids is crucial for its survival in the stomach, where it encounters fluctuating pH and digestive enzymes. Fatty acid biosynthesis inhibitors are being explored as a way to compromise *H. pylori* membrane integrity and survival [108].

Recent studies have shown that enzymes involved in fatty acid synthesis, such as acyl-CoA synthetases, are crucial for *H. pylori* growth and virulence. Targeting these enzymes with small-molecule inhibitors could impair membrane biogenesis and disrupt bacterial motility and adhesion. This would limit the ability of *H. pylori* to colonize the gastric mucosa, potentially reducing the severity of infection. However, development of these inhibitors is challenging due to mutation [109]. 

Modification of membrane lipids, mainly through the biosynthesis of cholesterol-6′-acyl-α-glucoside, can also reduce the adhesive capacity of *H. pylori*, thereby facilitating its eradication [110]. Targeting fatty acids in *H. pylori* can be effective in disrupting membrane functions, impairing its motility, and aiding in easier bacterial eradication.

#### 7.2.4. Purine Metabolism

Purine metabolism is a critical process for *H. pylori* growth and survival, as purines are essential for DNA and RNA synthesis. The bacterium has a unique purine salvage pathway, which allows it to absorb purines from its environment and host [111]. Inhibiting enzymes involved in purine biosynthesis or salvage, such as inosine-5′-monophosphate dehydrogenase (IMPDH), could significantly impair *H. pylori* growth [112].

IMPDH is a key enzyme in the purine biosynthesis pathway and has been identified as a potential target for drug development. Inhibitors of IMPDH, such as sulfonyl-α-l-amino acids or methylpyrazole-substituted benzimidazoles, have demonstrated activity against a range of pathogenic bacteria, including *H. pylori*, by reducing DNA and RNA synthesis and thereby limiting bacterial proliferation [112,113]. Further exploration of purine metabolism inhibitors could provide novel therapeutic options, particularly when used in combination with other antimicrobial agents to overcome antibiotic resistance [113].

#### 7.2.5. Metabolic Pathways of Iron in *H. pylori*

Iron is an essential nutrient for *H. pylori*, playing a crucial role in its growth and pathogenicity. The bacterium has developed sophisticated iron acquisition systems Feo system comprising feoA and feoB, which is crucial for it to extract iron from the host [114]. Iron acquisition is vital for *H. pylori* to thrive in the iron-limited environment of the stomach, where the host restricts iron availability as part of its immune defense [115].

FeoB receptors are highly specific for Fe^2+^ transport in *H. pylori*, and positive influence on their inhibition has been proven for N, N’-dicyclohexylcarbodiimide (DCCD), carbonyl cyanide-p-trifluoromethoxyphenylhydrazone (FCCP), and vanadate, resulting in impaired transport of iron, leading to inhibition of bacterial growth [116].

Studies have also been conducted on the effects of human lactoferrin (h-LF)—an iron-binding protein that limits the availability of iron in the environment where *H. pylori* resides. In response to infection, lactoferrin levels increase, serving as a defense mechanism within the body [117]. Lactoferrin exhibits numerous properties, including anti-inflammatory and antibacterial effects [118]. *H. pylori* colonizes the stomach, while lactoferrin undergoes proteolysis under the influence of gastric juice, leading to the formation of lactoferricin (h-LFcin)—a peptide with even stronger antibacterial activity than lactoferrin itself. Studies have shown that oral administration of lactoferrin may aid in the eradication of *H. pylori* [119].

## 8. Vaccine Against *Helicobacter pylori*

The validity of vaccinations against bacteria is confirmed by the effectiveness of currently available vaccinations such as meningococcal vaccine or vaccines against different bacteria mostly affecting the human respiratory tract [120,121]. The satisfactory effectiveness of vaccination against bacteria has prompted scientists to study the development of a vaccine against *Helicobacter pylori*. Studies are currently being conducted on several formulations as a potential vaccine. Targets for vaccine action include helicobacter virulence factors like urease, vacA, FlaA, AlpB, SabA, HpaA, and neutrophil-activating protein A subunit [yunle 2024]. Potential vaccine vectors that have been considered include microorganisms such as *Saccharomyces cerevisiae*, *Listeria monocytogenes* and *L. lactis* [122,123]. Since some researchers suggest that multivalent vaccines are more effective, there are two studies on this kind of vaccine targeting T- or B-cell epitopes [124]. In 2019, research on peptide vaccines was published. An immunogen-derived peptide antigen with specific amino acid sequence—MVTLINNE peptide—immunizes against *H. pylori* infection by mediating in increase in IgA production, crucial in the immune response during *H. pylori* infection [125]. 

DNA vaccines have many advantages over conventional ones; therefore, they are a point of interest for scientists. Some of their superiorities are greater safety, ease of development and production, ability to induce a wider range of immune response, and no risk of infection caused by vaccination [126]. Recent research has proved that a potential DNA vaccine construct can be based on virulence factor genes such as *cagW* and *flaA*, due to their important role in invasion [127,128].

Currently, all the research on a vaccine against *H. pylori* is at a very early stage. The only candidate admitted for the third phase of research in China has been withdrawn [129]. However, development of a vaccine against *H. pylori* should be continued to decrease the prevalence of *H. pylori,* especially to reduce the incidence of gastric cancer. There are some promising targets that could be used to create a vaccine. Moreover, further vaccine candidates have been proposed and evaluated in preclinical and early clinical studies. These include multicomponent subunit vaccines containing recombinant *H. pylori* antigens such as neutrophil-activating protein (NAP), CagA, and VacA, formulated with suitable adjuvants, which have demonstrated good immunogenicity profiles in phase I and II trials performed in Europe and North America. A prophylactic oral vaccine (UreB/LTB) has demonstrated moderate success (about 72%) in children, but broader, lasting protection remains a challenge [130]. Subcutaneous recombinant adhesion-antigen vaccines (BabA/B/SabA) induced high-antibody titres and significantly protected mice from gastric cancer with 100% efficacy in a therapeutic setting [131]. Animal studies offer promise, especially for multivalent and adhesion-based vaccines, but human translation is limited. Improving preclinical models (e.g., non-human primates) and identifying clear correlates of protection could build a bridge across the animal-to-human gap. Future success depends on more immunogenic platforms, tackling strain diversity, and precise immune targeting. Other approaches, such as vector-based vaccines or DNA vaccines targeting *H. pylori* virulence factors, are also under investigation in experimental models. Inspired by success in respiratory pathogens, for mucosal immune activation and durable memory T- and B-cell responses have been explored. Using advanced platforms like mRNA-LNP (mRNA delivered via lipid nanoparticles), better adjuvants to bolster mucosal and memory responses might improve the immunogenicity and durability of the vaccine. mRNA/LNP technology is being explored to deliver antigens such as UreB, VacA, CagA, or synthetic multi-epitope constructs. The could enable development of mucosal immunity (via oral/intranasal delivery), durable immune memory to prevent reinfection, and simplified production for multivalent vaccines. Future research should divide prophylactic targeting of children prior to colonization from therapeutic strategies aiming to clear infection or prevent cancer progression [132]. 

## 9. Conclusions

The increasing resistance of *Helicobacter pylori* strains, particularly to widely used antibiotics, poses a crucial challenge to current eradication strategies. This progressive resistance highlights the urgent need for novel therapeutic strategies and the exploration of new antimicrobial agents. Innovative antibacterial compounds that target previously unexploited pathways within the bacterial cell hold potential to overcome existing resistance mechanisms, such as efflux pumps or alterations to bacterial target structures. This review summarizes recent studies that have identified several promising targets and compounds with anti-*H. pylori* activity, including those interfering with cell membrane integrity or essential bacterial enzymes or metabolic pathways. These are promising avenues for improving *H. pylori* management through the deployment of alternative antimicrobials. Efforts to develop a vaccine against *H. pylori* remain in a preliminary phase with limited progress towards a viable prophylactic or therapeutic option. While initial results are encouraging, substantial research is still required to translate these innovations into clinically applicable solutions. Future efforts should prioritize the development of effective, safe, and cost-accessible treatments capable of addressing the evolving resistance landscape and improving patient outcomes on a global scale.
